# [Corrigendum] MicroRNA-663b enhances migration and invasion by targeting adenomatous polyposis coli 2 in colorectal carcinoma cells

**DOI:** 10.3892/ol.2026.15734

**Published:** 2026-06-29

**Authors:** Fenqiang Xiao, Wangbin Chen, Chao Yu, Gang Zhao

Oncol Lett 19: 3701–3710, 2020; DOI: 10.3892/ol.2020.11482

Subsequently to the publication of the above article, an interested reader drew to the authors’ attention that, regarding the wound healing assay data shown in Fig. 3 on p. 3706, the 36 h/miR-663b mimic’ data panel in Fig. 3A appeared to contain a small overlapping section with the 36 h/siAPC2’ data panel in Fig. 3C, suggesting that these data panels, which were intended to show the results from differently performed experiments, had apparently been derived from the same original source. In addition, regarding the Transwell invasion assay data shown in Fig. 4 on p. 3707, the ‘miR-663b mimics’ data panel in Fig. 4A contained an overlapping section with the ‘si APC2’ data panel in Fig. 4C, such that these data were also apparently derived from the same original source. A subsequent independent investigation of the data in this paper undertaken by the Editorial Office revealed that the western blots featured in Fig. 3D were strikingly similar to blots that had appeared in a previously published paper in the *Journal of Translational Medicine* written by the same research group.

After examining their original data, the authors have realized that Figs. 3 and 4 in their paper were inadvertently assembled incorrectly. The corrected versions of Fig. 3 (now showing the correct 36 h/siAPC2’ data panel in Fig. 3C, the correct western blot data in Fig. 3D and replacement data for the scratch wound assay experiments shown in Fig. 3B) and Fig. 4 (now showing the correct data for the ‘si APC2’ data panel in Fig. 4C) are shown on the next two pages. Note that the revisions made to these figures do not affect the overall conclusions reported in the paper. The authors are grateful to the Editor of *Oncology Letters* for allowing them the opportunity to publish this Corrigendum, and all authors agree with its publication. Furthermore, the authors apologize to the readership for any inconvenience caused.

## Figures and Tables

**Figure 3.**
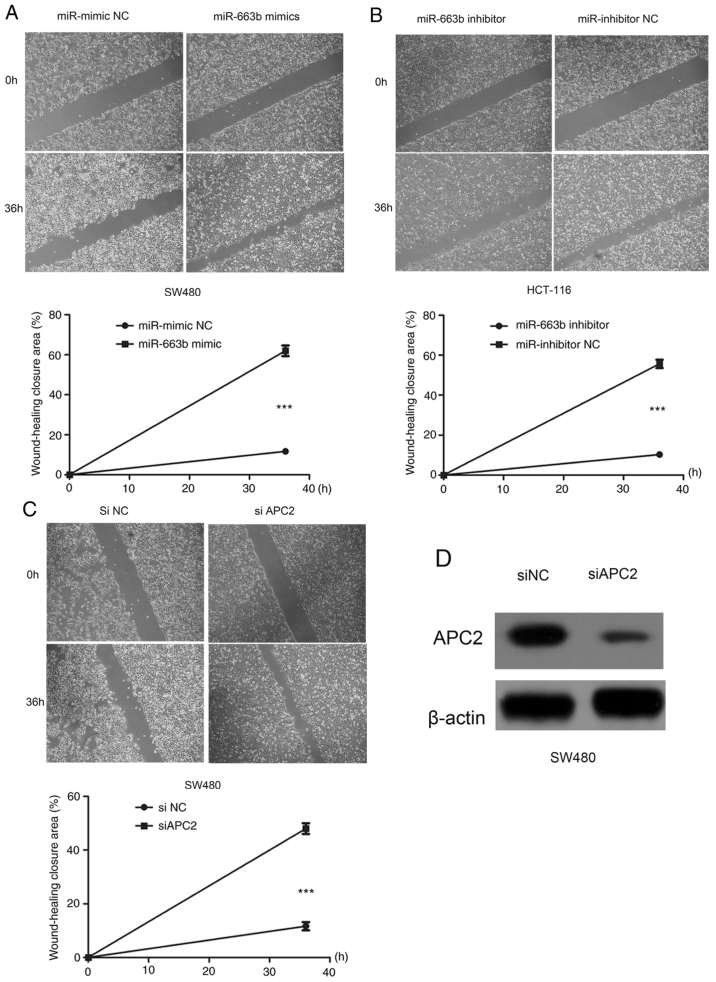
Effects of miR-663b and siAPC2 on migration of colorectal cancer cells were evaluated via a wound healing assay. (A) The ectopic expression of miR-663b promoted SW480 cell migration. (B) miR-663b knockdown inhibited HCT-116 cell migration. (C) APC2 knockdown elicited the same effect as ectopic miR-663b on migration. (D) siRNA targeting APC2 significantly downregulated the APC2 protein level. ***P<0.001 vs. NC. miR, microRNA; si, small interfering; APC2, adenomatous polyposis coli 2; NC, negative control. Magnification, ×40.

**Figure 4.**
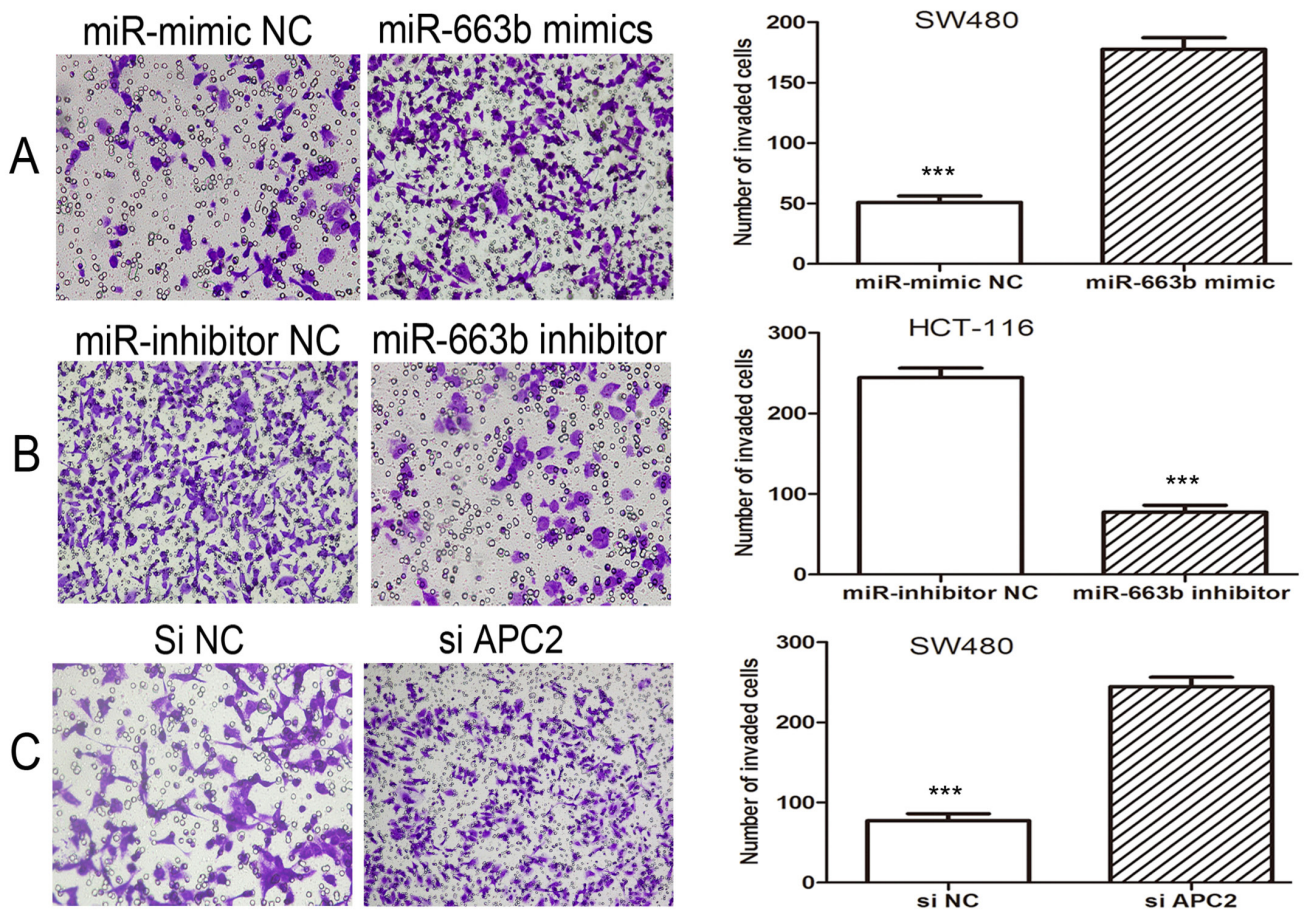
Effects of miR-663b and siAPC2 on invasion of colorectal cancer cells were evaluated via a Transwell invasion assay. (A) miR-663b overexpression significantly enhanced the invasive capacity of SW480 cells. (B) miR-663b knockdown inhibited invasion of HCT-116 cells. (C) APC2 knockdown elicited the same effect as ectopic miR-663b on invasion. Data are presented as the mean of 3 measurements and the bars represent the standard deviation. ***P<0.001 vs. NC. miR, microRNA; si, small interfering; APC2, adenomatous polyposis coli 2; NC, negative control. Magnification, ×100.

